# Acute Hormonal and Force Responses to Combined Strength and Endurance Loadings in Men and Women: The “Order Effect”

**DOI:** 10.1371/journal.pone.0055051

**Published:** 2013-02-07

**Authors:** Ritva S. Taipale, Keijo Häkkinen

**Affiliations:** Department of Biology of Physical Activity, University of Jyväskylä, Jyväskylä, Finland; University of Navarra School of Medicine and Center for Applied Medical Research (CIMA), Spain

## Abstract

**Purpose:**

To examine acute responses and recovery of serum hormones and muscle force following combined strength (S) and endurance (E) loading sessions in which the order of exercises is reversed (ES vs. SE).

**Methods:**

This cross-over study design included recreationally endurance trained men and women (age 21–45 years, n = 12 men n = 10 women) who performed both loadings. Maximal bilateral isometric strength (MVC), isometric rate of force development (RFD) and serum concentrations of testosterone (T), cortisol (C), growth hormone (GH), insulin-like growth factor 1 (IGF-1), binding protein 3 (IGFBP3) and sex hormone binding globulin (SHBG) were measured during and after both loadings.

**Results:**

Both of the present combined (ES and SE) loadings led to a greater acute decrease in MVC in men than in women, while RFD was slightly affected only in men. Recovery of MVC and RFD to baseline was complete at 24 h regardless of the order of exercises. In men, neuromuscular fatigue was accompanied by increased C concentrations observed post SE. This was followed by decreased concentrations of T at 24 h and 48 h that were significantly lower than those observed following ES. GH response in men also differed significantly post loadings. In women, only a significant difference in T between ES and SE loadings was observed at post.

**Conclusion:**

These observed differences in hormonal responses despite similarities in neuromuscular fatigue in men indicate the presence of an order effect as the body was not fully recovered at 48 h following SE. These findings may be applicable in training prescription in order to optimize specific training adaptations.

## Introduction

Hormones have an integral role in daily physiological function of humans. In large part, age, sex, and diurnal variations determine hormonal concentrations at rest, while physical and psychological stressors are known to stimulate unique responses. Physical stress, more specifically, exercise mode (strength training vs. endurance training), intensity [1], [2] and duration [3], [4] influence hormonal concentrations that initiate various physiological cascades that may contribute to muscle hypertrophy [5], increased capillary density [6] and initiation of mitochondrial biogenesis [7]. Rest/recovery after exercise [5] also plays a considerable role in maintaining homeostasis, while training status may augment or attenuate specific acute responses [1].

Testosterone is an important anabolic steroid that may increase remarkably as an acute response to both endurance [3] and heavy resistance exercise [5] Endurance exercise [8] and submaximal or explosive type resistance exercise do not always induce as great a response [2], while endurance exercise of long duration (e.g. 2 hours) typically decreases testosterone levels, while cortisol levels tend to increase [3]. Cortisol is a catabolic steroid that, in high concentrations, has been reported to interfere with the anabolic processes that promote muscle hypertrophy, which may in turn negatively affect strength development [9]–[11].

Growth hormone (GH) and insulin like growth factor-1 (IGF-1) work both synergistically and independently to stimulate many metabolic functions [12], [13]. Growth hormone (GH) is considered to be an anabolic “family of related polypeptides” [14] that stimulates protein synthesis, cell reproduction and renewal. IGF-1 is positively associated with parameters of good health including cardiovascular fitness and body composition [15]. GH concentrations generally increase in response to both strength and endurance exercise thereby simulating IGF-1 production.

Serum hormone concentrations are influenced by the availability of hormone transporters and receptors. Sex hormone binding globulin (SHBG) has a specific “high-affinity” binding site for transporting sex hormones like testosterone while IGF-1 binding protein 3 (IGFBP3) transports and helps to control the distribution of the majority of IGF-1 in circulation [16]. Binding of hormones to proteins/globulins allows for an extended period of transport throughout the body, thus increasing the potential for action.

It is well established that both strength and endurance exercise can cause acute fatigue resulting in reduced strength and power of loaded muscles. Both maximal and explosive type strength exercises are able to induce acute fatigue; however, the mechanisms behind this fatigue may differ [17]. An endurance training session does not cause as much fatigue as a strength training session due to the differences in force production. Typical strength exercises require high levels of muscle activation, while endurance training demands lower levels of repetitive force production and even maximal uphill running cannot evoke maximal muscle activation [18].

The benefits of strength training for endurance performance has been well documented in recent years (e.g. [19]–[21]), though interference has been observed under certain conditions [22]. Studies on the acute responses to combined strength and endurance exercise and the specific role of order are limited. Endurance exercise followed by strength exercise has been shown to influence the magnitude of the testosterone, but not the cortisol response in strength trained men [23]; however, the acute hormone responses to strength and endurance training in a combined session and changes in concentrations during the subsequent recovery have not yet been illuminated. It may be hypothesized that the order of combined exercise will induce fatigue that is specific and may differ in magnitude affecting the subsequent exercise session in terms of training effectiveness [24].

The purpose of this study was to examine the acute exercise-induced serum hormone and neuromuscular responses and the time course of changes during recovery (24 and 48 hours post loading) following a loading of combined strength and endurance training session in recreationally endurance trained male and female runners. One loading session started with endurance exercise, which was immediately followed by strength exercise (ES) while the other loading session started with strength exercise, which was immediately followed by endurance exercise (SE). The possibility of an “order effect” on these responses was examined using this cross-sectional design.

## Methods

### Subjects

Subjects with a recreational endurance running background (age 21–45 years, n = 12 men n = 10 women) were recruited to participate in this study (Table 1). The target group included healthy men and women, exclusion criteria included: body mass index >28 kg · m^−2^, illness, disease, injury or use of medications that would contraindicate participation in the study.

**Table 1:** Subject Characteristics.

		Men (n = 12)	Women (n = 10)
**Height**	**(cm)**	177.4±6.4	165.9±7.6
**Weight**	**(kg)**	75.7±3.6	59.8±5.1
**Fat**	**(%)**	12.9±3.6	22.0±3.8
**BMI**		24.1±1.3	21.7±1.8
**VO_2max_**	**(l/min)**	4.1±0.3	2.9±0.4
	**(ml/kg/min)**	54.5±4.0	48.5±4.6

### Ethics Statement

Ethical approval of methodology and consent procedures were granted by the University of Jyväskylä Ethical Committee and the study was conducted according to the provisions of the most recent Declaration of Helsinki. Subjects received written and oral information about the study design and measurement procedures. The possible risks and benefits of participation in the study were thoroughly explained prior to signing two copies of an informed consent document (one for the subjects’ records and the other for our records). A resting electrocardiogram and health history questionnaires filled out by each subject were reviewed and approved by a physician prior to participation in the study.

Prior to the specific loading sessions, subjects completed a set of tests to familiarize themselves with the strength training equipment and to determine appropriated loads/intensities for the specific loadings. Measurements included: maximal isometric bilateral leg extension force and rate of force development as well as maximal oxygen uptake (VO_2max_). Body composition and baseline resting blood samples for serum hormones were also measured. Following completion of these baseline tests, subjects performed, in random order, two loadings of strength and endurance exercise (ES or SE) which were completed in a single session. Approximately one to three weeks separated pre-testing and loadings. Time of day variations in force and hormonal variables were controlled for by making sure that each subject performed loadings ±1 hour from their pre-testing time and by taking blood samples in the morning (in a rested and fasted state) on each testing day.

#### The strength loading

focused primarily on the leg extensors and included both maximal and explosive strength exercises. Loads of 70–85% of 1RM were used for maximal strength exercises that included three sets of 5–8 repetitions. The final repetition of each set was near failure. Explosive strength exercises included three sets of 8–10 repetitions with maximal velocity of each repetition using 30–40% 1RM load. Exercises included: maximal bilateral leg press (3 sets maximal and 3 sets explosive), squat (3 sets maximal), loaded squat jump (3 sets explosive), and calf raises (2 sets maximal). Strength exercises were performed in a circuit such that leg press exercises were at the beginning, middle and end of the training session. There was 2 minutes of rest between sets. The total duration of strength training session was approximately 45 minutes**.**

#### The endurance loading

consisted of running on a 200 m indoor track. The intensity of running was at a steady-state at between each subject’s previously determined individual lactate threshold (LT) and respiratory compensation threshold (RCT) for 60 minutes.

### Body Composition

In addition to standing height, body mass and body composition were measured using bioimpedance (InBody720 body composition analyzer, Biospace Co. Ltd, Seoul, South Korea). Measurements were always taken in conjunction with morning blood tests between 07.30–08.00 and subjects always arrived for testing in a fasted state, thus helping to keep the possible confounding variables of diet and hydration status to a minimum. Subjects were measured in their undergarments.

### Aerobic Capacity

Endurance performance characteristics were measured using a treadmill running protocol [25]. The running velocity began at 8 km·h^−1^ and was increased by 1 km·h^−1^ every third minute until volitional exhaustion. Treadmill incline remained a constant 0.5 degrees throughout the test. Heart rate was recorded continuously using a heart rate monitor (Suunto t6, Vantaa, Finland). Mean heart rate values from the last minute of each stage were used for analysis. Oxygen consumption was measured breath-by-breath throughout the test using a portable gas analyzer (Oxycon Mobile®, Jaeger, Hoechberg, Germany). Maximal oxygen uptake (VO_2max_) was determined to the highest average 60 s VO_2_ value. Other factors such as a heart rate, VO_2_, and respiratory exchange ratio were monitored for determination of maximal effort. Fingertip blood samples were taken every 3rd minute to measure blood lactate concentrations. For blood sampling, the treadmill was stopped for approximately 15–20 seconds. Blood lactates were analyzed using a Biosen S_line Lab+ lactate analyzer (EKF Diagnostic, Magdeburg, Germany). Lactate threshold (LT) and respiratory compensation threshold (RCT) were determined using blood lactate, ventilation, VO_2_ and VCO_2_ (production of carbon dioxide) according to [26].

### Strength Measurements, Isometric Leg Press

An electromechanical isometric leg extension device (horizontal leg press, designed and manufactured by the Department of Biology of Physical Activity, University of Jyväskylä, Finland) was used to measure maximal bilateral strength and rate of force development (RFD). The subjects’ knee angle was 107° measured using the greater trochanter, lateral tibiofemoral joint space and lateral malleolus as reference points, while the hip angle was 110°. Full extension of the leg was measured as 180°. Subjects were instructed to produce force “as fast and as hard as possible” for approximately 3 s. Force data was collected at a sampling frequency of 2000 Hz, and then filtered (20 Hz low pass filter). RFD was assessed over 20 ms (±10 s from maximal rate of force development). Force data was analyzed using customized scripts (Signal 4.04, CED, UK). Subjects performed at least three maximum voluntary contractions [27]. If the maximum force during the last trial was greater than 5% compared to the previous trial, and additional trial was performed. The best performance trial, in terms of maximal force measured in newtons (N), was used for statistical analysis. The reliability of these measurement techniques has been previously reported [28]. The intra-class coefficient of variation for strength measurements for the present study was 5.1% with an intra-class correlation of 0.98.

### Blood Samples and Serum Hormones

Venous blood samples (10 ml) were collected after 12 hours of fasting between 07.30–08.00. Blood samples were collected using sterile needles into serum tubes (Venosafe, Terumo Medical Co., Leuven, Belgium) by a qualified lab technician who reviewed analyses of the basic blood count (Sysmex KX-21N, Kobe, Japan) to check for abnormalities prior to testing. Whole blood was centrifuged at 2500 g (Megafuge 1.0R, Heraeus, Germany) for 10 min after which serum was removed and stored at −80°C until analysis. Blood samples were used for determination of serum testosterone, cortisol, growth hormone, insulin-like growth factor 1 and sex hormone binding globulin. Analyses were performed using chemical luminescence techniques (Immunlite 1000, DCP Diagnostics Corporation, Los Angeles, California, USA) and hormone specific immunoassay kits (Siemens, New York, NY, USA). The sensitivity of testosterone, cortisol, growth hormone, IGF-1, SHBG and IGF binding protein 3 assays were: 0.5 nmol·l^−1^, 5.5 nmol·l^−1^, 0.026 mlU·l^−1^, 2.6 nmol·l^−1^, 5.5 nmol·l^−1^, and 0.1 µg ·ml^−1^, respectively. The intra-assay coefficients of variation for testosterone, cortisol, growth hormone, IGF-1, SHBG, and IGF-1 BP-3 were: 5.7, 4.6, 4.2, 3.1, 2.4, and 4.4% respectively.

### Statistical Methods

Standard statistical methods were used for calculation of means and standard deviation (SD). Group differences and group-by-loading interaction were analyzed by a repeated analysis using mixed models and an unstructured covariance matrix. Groups were compared with least significant difference (LSD) post hoc analysis in a mixed models analysis when appropriate. The criterion for significance was set at * = p = 0.05, ** = p<0.01 and *** = p<0.001. Statistical analysis was completed with PASW Statistics 18 (SPSS Inc., Chicago, IL, USA).

## Results

**Figure 1:**
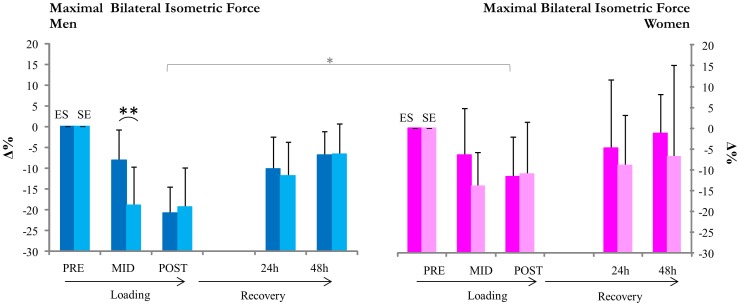
Maximal isometric force. **, *** inside column = significant change (p<0.01, p<0.001) in absolute maximal isometric force from pre,+++ = significant change (p<0.001) in absolute maximal isometric force from mid. **( = p<0.01 significant differences between relative changes from pre in ES and SE loadings.

**Figure 2:**
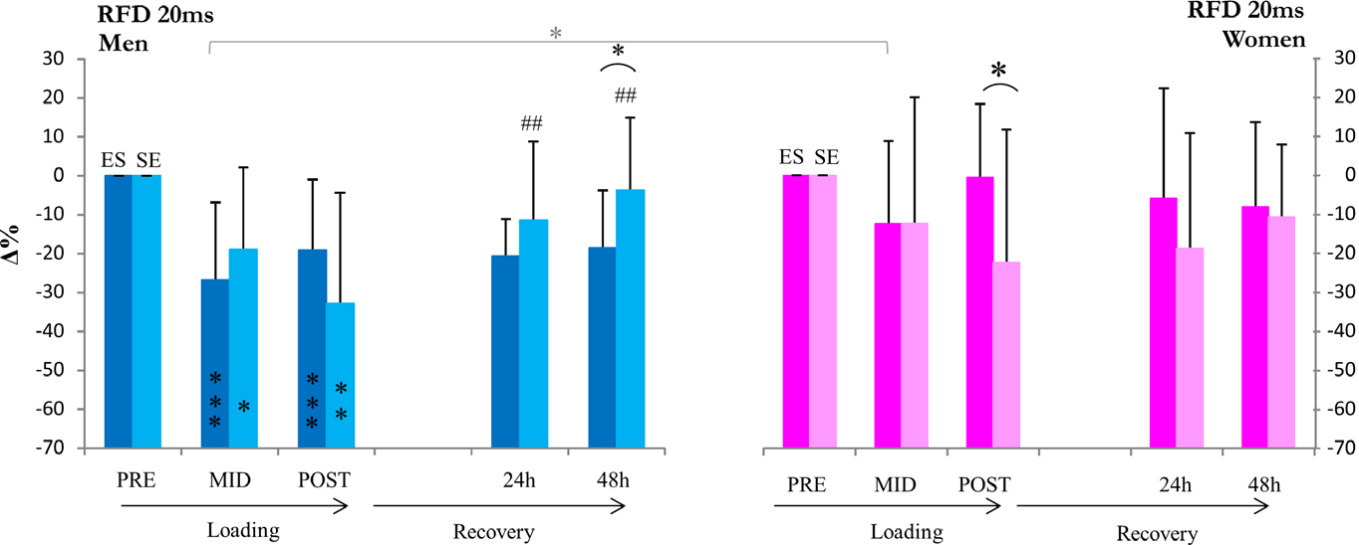
Isometric rate of force development. *, **, *** inside column = significant change (p<0.05, p<0.01, p<0.001) in absolute rate of force development from pre, ## = significant change (p<0.01) in absolute rate of force development from post. *[ = significant difference (p<0.05) between changes in RFD from pre in men and women. *( = significant difference (p<0.05) between relative changes from pre in ES and SE loadings.

Both ES and SE loadings led to decreases in maximal and explosive strength. Absolute maximal bilateral isometric (MVC) strength in ES men decreased significantly following E (−8±7%, p = 0.002 at MID) but not in women (Figure 1). After completion of both E and S, absolute strength decreased significantly in both men and women (−21±7%, p<0.001 and −12±9%, p = 0.007, respectively). In SE, absolute strength decreased significantly following S in both men and women (-19±9%, p<0.001 and −14±8%, p = 0.015). After both S and E had been completed, absolute strength remained significantly reduced in men, while only a statistical trend was observed in women (−19±9%, p<0.001 and −12±12% p = 0.074, respectively. The relative loading-induced decreases (Δ%) in strength between ES and SE differed significantly at mid in men (p<0.001) (Figure 1). The relative decreases (Δ%) in strength in men and women were similar at MID; however, at post, a significant difference between ES men and ES women was observed. The absolute rate of force development (RFD) decreased during both loadings in ES and SE men (at post −19±18%, p<0.001 and −33±28%, p = 0.003, respectively, Figure 2). In women, RFD did not decrease significantly (at post −1±19% in ES and −22±34% in SE). The relative RFD was statistically different between men and women at post in ES. While RFD had returned to baseline in both men and women following ES and SE, MVC remained significantly decreased in SE and ES of men at 24 h (p<0.01, −14% and p<0.001, −15%, respectively) and in ES of men at 48 h (p<0.01, −11%). No differences between loadings were observed.

**Figure 3:**
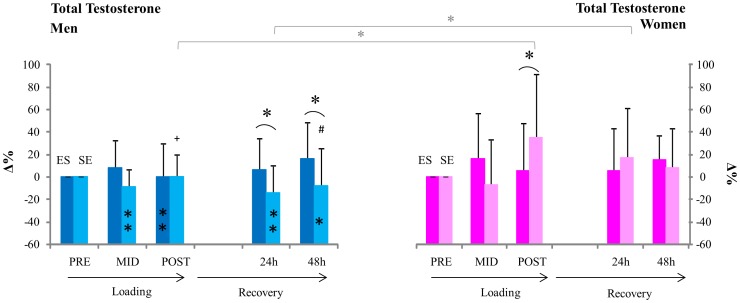
Serum total testosterone. ** inside column = significant change (p<0.01) from pre in absolute serum total testosterone concentrations,+ = significant change (p<0.05) in absolute serum total testosterone from mid. *[ = significant difference (p<0.05) between relative changes in serum testosterone concentrations from pre in men and women. *( = significant difference (p<0.05) between relative changes from pre in ES and SE loadings.

The relative change in testosterone concentrations of men from pre were similar at mid and post of ES and SE, while at 24 h and 48 h of recovery the relative change from pre in SE was significantly lower than the relative change from pre in ES (p<0.05 at 24 h and 48 h, Figure 3). The relative changes in serum testosterone concentrations in women were similar from pre to mid, but the change in SE was significantly greater than in ES at post (p<0.05). At 24 h and 48 h of recovery in women, the relative changes from pre in both ES and SE were similar. Significant differences in the relative responses of testosterone between men and women were observed; testosterone increased more significantly in women in comparison to men at post SE (p<0.05). ES men had significantly lowered testosterone in comparison to women (p<0.05) at 24 h of recovery. Significant differences in absolute serum testosterone concentrations were also observed between ES and SE in men at 24 and 48 h.

**Figure 4:**
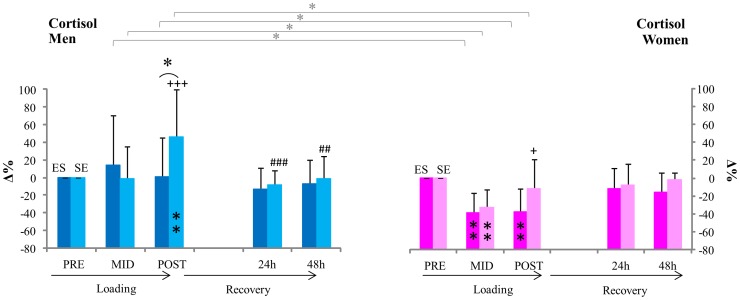
Serum cortisol. ** inside column = significant change (p<0.01) from pre in absolute serum cortisol concentrations,+++ = significant change (p<0.001) in absolute serum cortisol from mid. *[ = significant difference (p<0.05) between relative changes in serum cortisol concentrations from pre in men and women, *( = p<0.05 significant differences between relative changes from pre in ES and SE loadings.

The relative cortisol response from pre in men was significantly different between ES and SE at post (p<0.05), but did not differ at mid (Figure 4). In women, no significant differences in changes were observed between loadings. The magnitude of cortisol response between men and women was significantly different at both mid and post in both ES and SE (p<0.05). A significant difference in absolute serum cortisol of men was observed between ES and SE at post (p<0.05).

**Figure 5:**
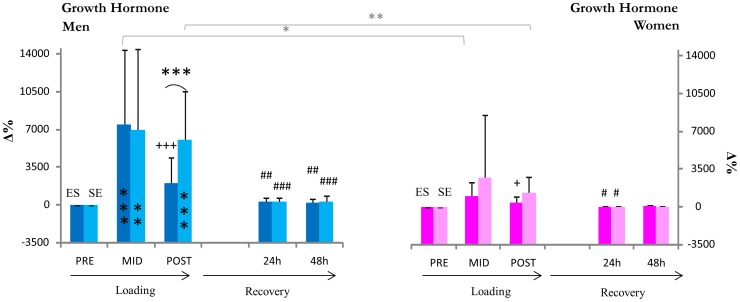
Growth hormone. **,*** inside column = significant change (p<0.01, p<0.001) from pre in absolute GH concentrations,+++ = significant change (p<0.001) in absolute GH from mid, ##, ### = significant change (p<0.01, p<0.001) in absolute GH from post, *[, **[ = significant difference (p<0.05, p<0.01) between changes in GH concentrations from pre in men and women, ***( = p<0.001 significant differences between relative changes from pre in ES and SE loadings.

In men, the magnitude of growth hormone increase observed between pre and post was significantly greater following SE than ES (p<0.001, Figure 5) while the magnitude of increase in GH from pre to mid of both loadings was similar. GH at 24 and 48 h had returned to baseline. In women, GH concentrations remained statistically unaltered. Difference in the magnitude of growth hormone responses between men and women at mid and post in both ES and SE were significant (p<0.05), while the difference between both ES and SE responses in men was greater (p<0.01) than in women. A significant difference in men of absolute GH concentrations was observed between ES and SE at post (p<0.01).

**Figure 6:**
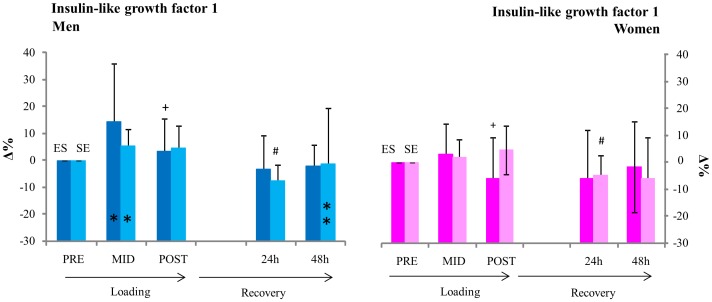
Insulin like growth factor 1. * inside column = significant change (p<0.05) from pre in absolute GH concentrations,+ = significant change (p<0.05) in absolute IGF-1 from mid, # = significant change (p<0.05) in absolute IGF-1 from post.

No statistically significant differences in relative changes of insulin-like growth factor 1concentrations were observed between men or women during or between ES and SE loadings (Figure 6).

A significant increase in absolute concentrations of insulin-like growth factor binding protein 3 was observed in ES men at mid (p<0.001) and post (p<0.05) loading (Table 2). A significant decrease back to baseline was observed in IGFBP3 from post to 24hours. Following the SE loading in men, a significant decrease in IGFBP3 from post to 48hours was observed (p<0.05). In ES women, a significant decrease in IGFBP3 was observed from mid to post (p<0.001) while in SE women, a significant decrease from the peak in IGFBP3 levels between post and 24 h was observed. No differences were observed between men and women in absolute IGFBP3 response; however, the magnitude of change in IGFBP3 was significantly different between ES men and women at post (p<0.05). Concentrations of serum SHBG remained statistically unaltered during the loading and recovery (Table 2).

**Table 2:** Absolute concentrations of insulin-like growth factor 1 binding protein 3 and sex-hormone binding globulin.

IGF BP 3 ( µg•ml^−1^)	PRE	MID	POST	24 h	48 h
Men ES	4.7±1.2	5.1±1.1 ^**^	5.1±1.1 ^*^	4.6±0.8 ^#^	4.6±0.7
Men SE	4.7±0.9	5.0±1.01	5.1±1.2	4.6±0.8	4.6±0.9 ^#^
Women ES	4.9±0.7	5.1±0.5	4.7±0.6 ^+++^	4.7±0.51	4.9±0.4
Women SE	4.8±0.8	4.8±0.7	5.1±1.0	4.6±0.6 ^#^	4.8±0.5

SHBG (nmol•l^−1^)	PRE	MID	POST	24 h	48 h
Men ES	35.9±9.2	37.4±9.1	37.2±9.1	34.0±8.5	35.7±9.0
Men SE	35.6±7.7	37.1±7.8	38.0±9.1	34.9±7.8	34.9±8.8
Women ES	73.9±24.9	76.4±28.9	73.5±24.7	72.4±27.1	74.2±21.6
Women SE	69.0±16.8	72.2±20.7	75.2±17.9	69.7±18.3	70.1±11.2

Significant difference from pre  =  ^*^, ^**^ (p<0.05, p<0.01). Significant difference from mid  =  ^+++^

(p<0.001). Significant difference from post  =  ^#^ (p<0.05).

Small changes were observed in absolute serum concentrations of hormones measured in the morning over the 3-day intervention (Table 3). Serum testosterone differed between ES and SE at recovery of 24 h after loadings in men due to a significant decrease in morning serum testosterone observed in SE. At 48 hours, serum concentrations of testosterone in SE men had returned to baseline. No changes were observed in morning concentrations of testosterone in women. Serum cortisol remained unaltered in men, while a significant decrease was observed in ES of women from pre-AM to 48 h-AM (p<0.01). These decreases in cortisol lead to a significant difference between loadings in ES and SE women.

**Table 3:** Absolute morning concentrations of serum hormones.

Testosterone (nmol•l^−1^)	PRE - AM	24 H - AM	48 H - AM
Men ES	16.0±4.2	15.5±3.4 ^¤^	15.7±4.1
Men SE	15.8±2.9	13.8±2.4 ^**, ¤^	14.5±5.1
Women ES	1.2±0.5	1.2±0.5	1.2±0.5
Women SE	1.1±0.4	1.0±0.2	1.1±0.3
**Cortisol (nmol•l** ^−**1**^ **)**			
Men ES	480±99	502±113	485±109
Men SE	505±121	479±83	489±90
Women ES	478±70	453±89	386±128 ^**. +, ¤^
Women SE	449±62	429±61	477±64^+, ¤^
**Growth Hormone (mlU•l** ^−**1)**^	**PRE - AM**	**24** **H - AM**	**48** **H - AM**
Men ES	0.41±0.33	0.48±0.40	0.92±1.45
Men SE	0.72±1.05	1.00±1.38	1.15±1.29^+^
Women ES	8.84±13.21	3.04±6.55 ^**^	1.80±1.49
Women SE	4.68±5.19	2.27±2.96 ^*^	2.68±3.16
**Insulin-like growth factor 1 (nmol•l** ^−**1**^ **)**			
Men ES	18.7±5.8	17.5±4.6	17.4±3.9 ^¤¤^
Men SE	20.0±4.7	20.2±6.5	21.9±6.5 ^¤¤^
Women ES	21.3±4.5	20.5±5.5	20.0±5.4
Women SE	20.7±5.4	20.1±5.3	20.5±5.3
**Insulin-like growth factor binding protein 3 (µg•ml** ^−**1**^ **)**	**PRE - AM**	**24** **H - AM**	**48** **H - AM**
Men ES	4.9±1.7	4.6±0.8	4.6±0.7
Men SE	4.9±1.15	4.6±0.8	4.6±0.9
Women ES	4.9±0.5	4.7±0.5	4.9±0.4^+^
Women SE	4.8±0.8	4.6±0.6	4.8±0.5
**Sex hormone binding globulin (nmol•l** ^−**1**^ **)**			
Men ES	34.7±10.1	34.0±8.5	35.7±9.0
Men SE	35.1±8.1	34.9±7.8	34.9±8.8
Women ES	72.9±26.4	72.4±27.1	74.2±21.6
Women SE	69.1±16.1	69.7±18.3	70.11±11.2

Significant difference between loadings = ¤ (p<0.05) and ¤¤ (p<0.01). Significant difference from Pre – AM measurements = * (p<0.05) and ** (p<0.01. Significant difference from 24 h – AM measurements = + (p<0.05). Significant difference between men and women in T at all time-points, in GH at pre in S+E p<0.05 and in SHBG at all time-points.

No significant changes were observed between the loadings in GH of men or women. Insulin-like growth factor-1 concentrations did not significantly fluctuate, however a significant difference between concentrations of IGF-1 was observed between ES and SE men at 48 h (p<0.01). Morning IGFBP3 concentrations in men remained statistically unaltered throughout the experimental period in men, whereas a significant increase in IGFBP3 was observed in ES women between 24 h-AM and 48 h-AM. Sex hormone binding globulin concentrations remained statistically unaltered.

## Discussion

The order in which single-session combined strength and endurance training is performed, either endurance followed by strength (ES) or strength followed by endurance (SE), appears to affect hormonal and neuromuscular responses in recreationally endurance trained men and women. In men, serum cortisol (C) response was significantly greater in SE than ES at post loading while testosterone concentrations during the present loadings (pre-post) remained relatively stable. At 24 h and 48 h of recovery, however, serum testosterone (T) concentrations in SE men were significantly lower than at baseline while also being lower than those of ES men at 24 h and 48 h. Significant differences in serum growth hormone (GH) responses of men were observed at post loading with concentrations remaining higher in SE despite the initial response to the first part of both ES and SE loadings being the same at mid. In women, a significant increase was observed in T at post SE whereas GH responses, though smaller, paralleled those of the men. Fatigue, as measured by maximal and explosive force, was the same in SE and ES post loading, but greater in men than in women. These responses suggest that performing strength training prior to endurance training is a more potent stimulus for hormonal responses, especially in men, than performing endurance training prior to strength training. The different responses to ES and SE loadings suggest that the order of exercises in single-session combined strength and endurance training should be considered even in recreational athletes because these responses may ultimately have physiological relevance in optimizing training.

The overall cumulative neuromuscular fatigue resulting from the present combined loadings was the same in SE and ES post loading, but fatigue was greater in men than in women. Both maximal isometric strength (MVC) and rate of force development (RFD) were significantly decreased at post, but appeared to recover at a similar rate following the loadings, despite remaining significantly decreased in men at 24 h and 48 h. Muscle activation was not examined in this study, thus it is unknown if, and to what extent, central fatigue along with the observed peripheral fatigue, may have been a factor. Nevertheless, based on previous research we can assume that maximal muscle activation decreased along with maximal strength [29]. While decreased force production is used as an indicator of fatigue, the time-course of these responses may be different from those observed in serum hormone concentrations that are used as biomarkers to assess physiological stress. In this investigation, primarily T, C, and GH seemed to be affected by order of exercise, whereas insulin-like growth factor 1 (IGF-1), insulin-like growth factor 1 binding protein 3 (IGF-BP3) and sex hormone binding globulin (SHBG) appeared to behave similarly regardless of the order of exercise.

An acute increase in T concentrations is a typical observation following a strength training session that has been linked to strength development and muscle growth after training (e.g. [5], [30]). In the present study, the T response in men was similar from pre to post in ES and SE, while in women a difference in the magnitude of change in T concentrations was observed at post ES and SE with a greater increase occurring following SE. In men, the T responses in ES and SE differed significantly during recovery at 24 h and 48 h post loading with relative T concentrations being lower on both days in SE than in ES. In women, T concentrations were similar at 24 h and 48 h of recovery. It appears that while voluntary force returned to baseline, serum hormone concentrations continued to respond to the exercise stimulus. In some studies, T has fully recovered after 1–2 days of rest [31], [32] whereas in other studies with very stressful resistance loading conditions, T levels remained decreased even after 2 days of rest [33]. This decreased T concentration may indicate that the hormone is being used for physiological processes or may be suggestive of the presence of a protein catabolic state [34], which may be undesirable should it persist for several days. It should be noted, however, that the decreased T observed in the present study was not accompanied by significantly elevated cortisol during recovery. Serum C concentrations in men were similar at mid ES and SE loadings, but significantly different at post, with a greater concentration of C observed in SE. Like other studies, the changes in C concentrations paralleled those observed in GH concentrations [4]. In women, there were no differences between ES and SE in terms of C behavior, but C was observed to decrease during the loading, a finding similar to Copeland et al. 2002 [35] who examined separate strength and endurance loadings in women. Recovery of C at 24 h and 48 h was complete in both loadings in men and women. As C plays an important role in activation of cascades leading to mitochondrial biogenesis in skeletal muscle [7], the greater magnitude of increase in C observed in SE of men and similar increase in women could, if it occurs with repeated training, be of physiological significance.

Previous studies have indicated that the magnitude of GH response is greater in “more fatiguing” strength training sessions [36], [37] and amplified with repeated exercise bouts of strength training [38]. This response is of interest because the initial GH response in this study following E and S at mid in men was the same, whereas GH concentrations decreased significantly when E was followed by S (ES) and remained elevated when S was followed by E (SE) at post. Strikingly similar responses were observed in GH response by Goto et al. 2005 [39] who examined the effects of a 60 minute low-intensity cycling protocol on a strength training session designed to induce pronounced hormonal responses [40]. In women GH responses were minimal, and recovery to baseline was complete in both men and women by 24 h. The reason behind the continued elevation in GH of men during the SE loading is difficult to explain, but may be linked to previous observations that GH concentrations begin to increase at the start of aerobic exercise with peak concentrations at or close to the end of aerobic exercise (e.g. [41]). The decrease in GH concentrations observed after mid in ES may be explained, in part, by the same phenomena because aerobic exercise was stopped and the overall intensity of the present strength loading was relatively low. It is important to remember that the present strength loading was made up of both maximal and explosive exercises, and included several periods of rest between each set of exercises. Lower volume strength and power loadings are known to induce a smaller hormone response than high volume loadings with short periods of rest [42]. As the GH variant examined in this study was the commonly examined and most abundant of GH variants (22 kDa variant), it is important to acknowledge that other GH variants may have responded differently and that it is necessary to exercise some caution when interpreting these results. It seems, however, reasonable to suggest that the extended period of GH elevation induced by SE may have physiological significance should it be present during repeated SE training sessions.

No differences were observed between ES and SE in IGF-1 responses and responses in men and women were not significantly different, these findings are parallel with Copeland et al. 2002 (women with 30 min recovery) [35]. IGF-1 and IGF-BP3 behaved similarly in ES and SE loadings and recovery in both men and women with a significant difference in magnitude of change observed only at post ES between men and women. SHBG was statistically unaltered during the loadings with no differences in responses observed between men and women. Our results parallel those of previous studies in which IGF-I levels were not affected in the 2 days following the loading protocols despite an increase in GH in men [13].

The difference observed between the acute responses of GH and IGF-1 supports the idea that these hormones work independently in response to exercise stress [13]. As blood samples were only taken post loading and some of the hormones e.g. GH are secreted in a pulsatile manner, we were unable to determine if there were further responses in the hours immediately after a training session as has been observed in other studies (e.g. [36]). In previous studies, however, GH concentrations appear to stabilize 1–2 hours after fatiguing heavy-resistance protocols [33].

Concentrations of serum hormones measured in the morning also appeared to be affected by loadings. In men, a significant decrease in T from baseline was observed after SE at 24 h-AM. The difference between ES and SE concentrations at this time point was significant and similar to that observed when examining corresponding time of day hormonal concentrations. These parallel changes in morning and time of day concentrations of T indicate that the SE exercise stimulus produced a greater stress response than ES and they correspond to findings that T may not fully return to resting values after training that induces physiological stress [33]. As testosterone levels typically increase overnight, this observation suggests that pituitary-testicular action was indeed suppressed. Following SE in women, a significant increase in morning C concentration was observed between 24 h-AM and 48 h-AM while a significant decrease in morning C concentration was observed in ES of women between 24 h-AM and 48 h-AM resulting in a significant difference between ES and SE, a finding that does not parallel time of day concentrations. This delayed C response could indicate that women did not recover completely from SE within the two days of rest examined.

The magnitudes of hormonal responses in women observed in the present study were somewhat smaller than those observed in men. This response was expected, as numerous studies have previously shown that hormonal responses in women are typically more subtle than in men [2], [40], [43], [44]. When studying women it is natural to contemplate the potential influence of different phases of the menstrual cycle, however, it appears that e.g. pituitary-adrenal responses to short-term, moderate intensity exercise are not significantly influenced by the menstrual cycle phase [45]. When examining acute hormonal responses to exercise in both men and women, circadian rhythms should also be taken into account. Previous studies have shown a substantial decrease in e.g. testosterone concentrations in the morning between 04∶00 and 08∶00 and a more gradual decrease from 08∶00 until 22∶00 [46], while others have shown evidence that cortisol is affected by diurnal variations in both young men and women [40], [47]. These diurnal variations, including nocturnal responses, may conceal some physiological responses as Häkkinen et al. [40] has postulated, on the other hand, e.g. the magnitude of growth hormone response to exercise appears to be independent of time of day [47]. In addition to diurnal variations, nutrition, overtraining/excessive fatigue and stress [5] as well as individual training status [48] can contribute to hormonal changes reported in studies. Taking this into consideration, the present study included a fairly homogenous group in terms of training status as well as specific instructions for subjects on how to prepare for each loading to minimize possible confounding factors. As the above factors have been taken into consideration, it may be suggested that the observed changes and differences in serum concentrations of hormones, though small, may have a dynamic role in subsequent physiological processes and training adaptations. In fact, there is some evidence that performing endurance training prior to strength training (ES) is more effective in improving endurance performance characteristics than SE training and E and S performed alone [49], [50], whereas intrasession sequence of E and S training may not influence adaptations in muscle strength and power [51].

### Conclusions

Both of the present combined (ES and SE) loadings led to more fatigue in men than in women in maximal bilateral isometric strength and isometric rate of force development was affected only in men, though to a smaller degree. In men, neuromuscular fatigue was accompanied by an increase in cortisol concentrations observed at post in SE, which was followed by decreased concentrations of testosterone in SE at 24 h and 48 h post loading. Despite the initial response to the first part of both ES and SE loadings being the same at mid, GH response in men was also observed to be different with concentrations remaining higher at post in SE. These observed differences in hormonal responses in men regardless of similarities in neuromuscular fatigue indicate the presence of an order effect. Acute hormonal responses in men in general were greater than in women. An order effect was also observed in women as a significant difference in testosterone between ES and SE loadings was observed at post, while no changes or order effect was observed during recovery. Although a great decrease in maximal strength was observed post loading in both SE and ES of men and women, there was no order effect observed in neuromuscular measures of either maximal or explosive strength. The present study does, however, demonstrate an order effect in hormone responses, particularly in T of men that indicated that the body may not have been fully recovered 24 h and 48 h following SE. The time-course of neuromuscular fatigue and recovery appears not to match the time-course of serum hormone responses to ES and SE loadings and an order effect appears to be present when combining strength and endurance training into a single session. These findings may be utilized in order to optimize training.
